# Carryover effects of feeding bulls with an omega-3-enriched-diet—From spermatozoa to developed embryos

**DOI:** 10.1371/journal.pone.0265650

**Published:** 2022-03-24

**Authors:** Dorit Kalo, Dan Reches, Noam Netta, Alisa Komsky-Elbaz, Yoel Zeron, Uzi Moallem, Zvi Roth

**Affiliations:** 1 Department of Animal Sciences, Robert H. Smith Faculty of Agriculture, Food and Environment, The Hebrew University, Rehovot, Israel; 2 ASRC, Animal Sperm Research Center, Department of Animal Sciences, Robert H. Smith Faculty of Agriculture, Food and Environment, The Hebrew University, Rehovot, Israel; 3 Department of Ruminant Science, Institute of Animal Science, Volcani Center, Bet-Dagan, Israel; 4 SION Artificial Insemination and Breeding Center, Hafetz-Haim, Israel; Universite Clermont Auvergne, FRANCE

## Abstract

The impact of omega-3 nutritional manipulation on semen cryosurvival and quality post thawing is controversial. Our aim was to examine how feeding bulls with omega-3 supplementation from different sources affects the spermatozoa quality parameters. Fifteen Israeli Holstein bulls were fed for 13 weeks with a standard ration top-dressed with encapsulated-fat supplementation: fish or flaxseed oil or saturated fatty acids (control). Ejaculates were collected before, during, and after the feeding trial. Frozen–thawed samples were evaluated by a flow cytometer for spermatozoa viability, mitochondrial membrane potential, the level of reactive oxygen species (ROS), acrosome membrane integrity, DNA fragmentation, phosphatidylserine translocation, and membrane fluidity. Both fish and flaxseed oil treatment resulted in lower ROS levels vs. control groups, during and after the feeding trial. Fewer spermatozoa with damaged acrosomes were observed in the fish oil group after the feeding trial. The spermatozoa membrane fluidity was altered in both the fish and flaxseed oil groups throughout the feeding trial, but only in the flaxseed oil group after the feeding trial. The proportion of spermatozoa with fragmented DNA was lower in the flaxseed oil group after the feeding trial. The spermatozoa fertilization competence did not differ between groups however, blastocyst formation rate was higher in the fish and flaxseed oil groups relative to the control. This was associated with differential gene expression in the blastocysts. Overall, the omega-3-enriched food improved the spermatozoa characteristics; this was further expressed in the developing blastocysts, suggesting a carryover effect from the spermatozoa to the embryos.

## Introduction

Nutrition manipulation has been shown to improve bull welfare [1] and to induce a beneficial impact on semen quality. Feed enrichment with polyunsaturated fatty acids (PUFAs) altered the fatty acid composition of bull spermatozoa and improved the semen quality [2]. Among the PUFAs, the essential fatty acids cannot be synthesized *de novo* and need to be supplemented in the diet. For instance, omega-3 and omega-6 families, which make up 30–50% of the total fatty acid content of the mammalian spermatozoa membrane [3], can be found in vegetable oils such as flaxseed oil and soybean oil, eggs, fish oil, seafood, and more [4]. Consumption of omega-3 as a nutraceutical product has been found to affect semen in stallions [5], boars [6, 7], bovine [8, 9], rams [10], and humans [11, 12]. In rams, dietary intake of fish oil increased the ejaculate concentration and the proportion of motile spermatozoa [13–15], as well as the proportion of normal spermatozoa with intact acrosome [15]. Feeding pigs with tuna oil increased the proportion of progressively motile spermatozoa and reduced the proportion of spermatozoa with abnormal morphology [16]. In addition, feeding young male goats with a fish oil-enriched diet improved testes development, reflected by the increased seminiferous tubule diameter and the number of Leydig and Sertoli cells, spermatogonia, spermatocytes, and spermatid cells [17]. Feeding Friesian bulls with flaxseed oil for 7 weeks improved the semen characteristics, as reflected in a higher percentage of live and motile spermatozoa, a higher concentration of spermatozoa, and a lower proportion of spermatozoa with abnormal morphology [18]. Omega-3 supplementation for 12 weeks to Holstein bulls exposed to heat stress improved the motility parameters of their fresh semen, with no effect on the frozen–thawed semen [9]. Feeding a flaxseed oil-enriched diet to Holstein bulls increased the spermatozoa progressive motility and velocity in fresh semen [8]. In contrast, in a recent study, PUFA enrichment of diets fed to young Nellore bulls for 10 months had a negative impact on their spermatozoa quality characteristics, including motility, membrane integrity, and lipid proportions [19].

The spermatozoa membrane composition is critical for capacitation [20], acrosome reaction [21], and spermatozoa–oocyte fusion through fertilization [22]. In mammalians the spermatozoa membrane is rich in PUFA, mainly docosahexaenoic acid and docosapentaenoic acid [22]. The membrane also contains high amounts of cholesterol and sphingomyelin, which are involved in membrane fluidity regulation [23]. A previous study suggested that PUFA supplementation can change the proportion of membrane phospholipids, thereby affecting the spermatozoa functionality [24]. This nutritional approach might be highly relevant for intensive reproductive management, i.e., artificial insemination, in which cows are inseminated with cryopreserved semen from high merit bulls [25]. Through cryopreservation, spermatozoa undergo physiological and structural alterations in their membrane composition, which might affect their viability and functionality [26, 27]. In light of this, it was resonable to hypothesize that feeding bulls with flaxseed oil or fish oil would change the membrane fatty acid compositionand would consequently improve spermatozoa cryosurvivel. In our previous study, we reported that feeding bulls with flaxseed oil or fish oil for 13 weeks increases the proportion of docosahexaenoic acid, α-linolenic acid, and total omega-3 fatty acids in fresh semen samples [8]. In the current study, using the same samples [8], we examined the effects of omega-3 supplementation on the spermatozoa viability and characteristics post thawing. In addition, we examined spermatozoa fertilization competence *in vitro*. We hypothesized that omega-3 supplementation would improve the spermatozoa cryosurvival and further affect fertilization capacity and embryonic development. The findings reported herein provide new information and enhance our understanding regarding omega-3 supplementation and its impact on cryopreserved-spermatozoa characteristics and fertilization capacity. Moreover, the study provides new evidence for paternal carry over effects from the spermatozoa to the developing blastocyst.

## Materials and methods

All chemicals were purchased from Sigma-Merck (Rehovot, Israel) unless otherwise indicated. All culture media were prepared in the laboratory as previously reported [28, 29].

### Feeding groups

**Fig 1:**
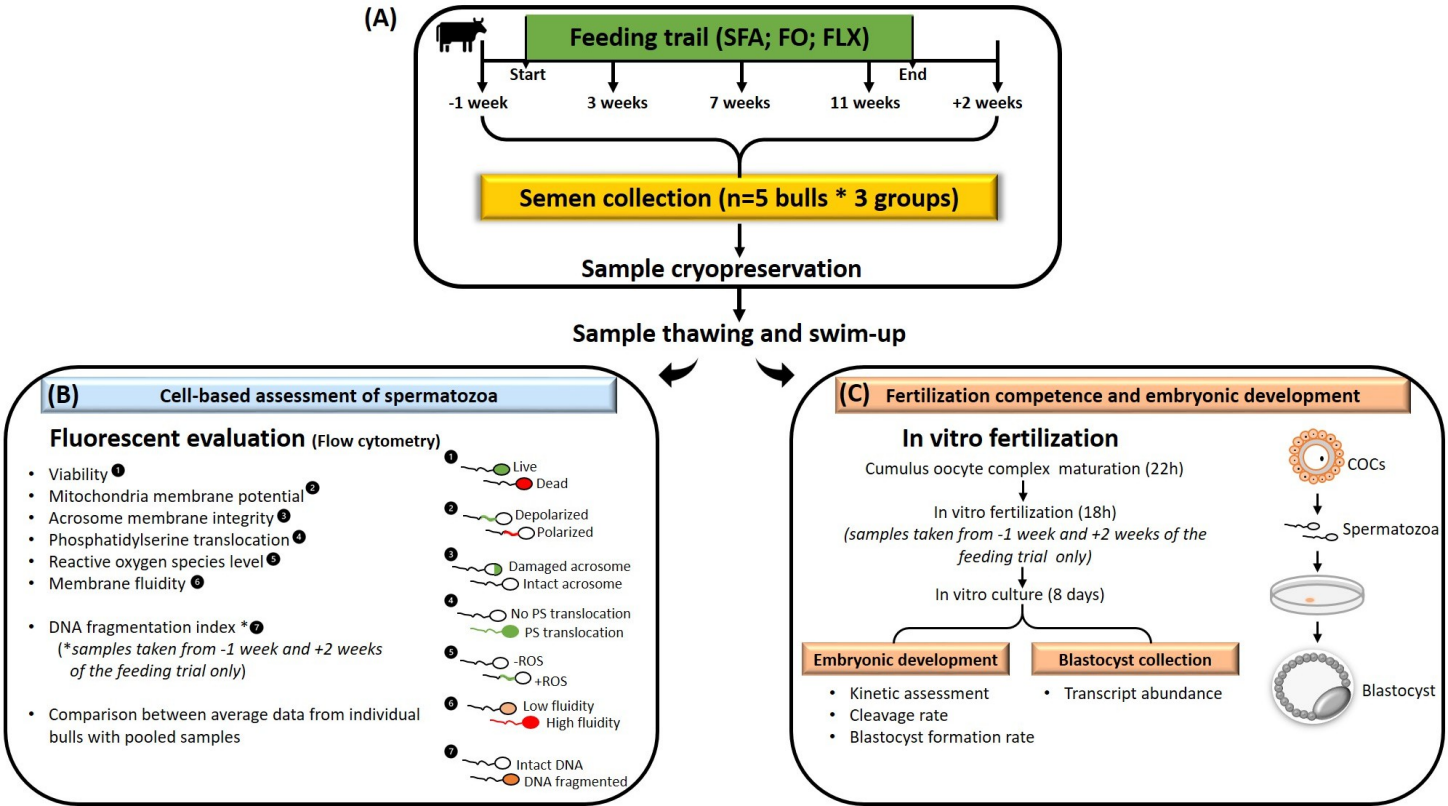
A schematic illustration of the experimental design. (A) Bulls (n = 15) were fed for 13 weeks (feeding period) with saturated fatty acid (SFA; control) or fish oil (FO) or flaxseed oil (FLX), as previously described by our group [8]. Semen was collected before (-1 week), during (3, 7, and 11 weeks) and after (+2 weeks) the feeding period. Collected semen was frozen in straws and stored in liquid nitrogen until further analysis. Samples were thawed, subjected to the swim-up procedure, and then evaluated for cell-based assessment of spermatozoa (B) and fertilization competence and embryonic development (C). (B) Cell-based assessment of spermatozoa (n = 25,000 spermatozoa/group) was evaluated by flow cytometry and included plasma membrane integrity, mitochondrial membrane potential, acrosomal membrane integrity, phosphatidylserine translocation, reactive oxygen species (ROS) level, membrane fluidity and DNA fragmentation index. (C) Fertilization competence and embryonic development were examined by *in-vitro* production of embryos. Cumulus oocyte complexes (COCs) were divided into three groups and *in-vitro* matured for 22 h, then fertilized with pooled SFA, FO, or FLX samples collected before the feeding period (-1 week; 3 replicates) and after the feeding period (+2 weeks; 3 replicates). Fertilization competence was recorded as the proportion of oocytes that cleaved to 2- to 4-cell-stage embryos 42–44 h post fertilization and the proportion of embryos that developed to the blastocyst stage on day 8 post fertilization. The embryos’ developmental kinetics was recorded in a time-lapse incubator, including the timing of the 1^st^, 2^nd^, and 3^rd^ division, and blastocyst formation (i.e., the formation of a blastocoel cavity within the embryo). Twelve blastocysts were individually collected from each experimental group and subjected to mRNA extraction, followed by qRT-PCR, to examine the long-lasting effects of treatment from the spermatozoa to the developed embryo (i.e., the paternal effect). Fig 1 was created by Dr. Kalo with Microsoft PowerPoint software.

Ejaculates were collected in our previous feeding trial as described by Moallem *et al*. (2015) and as presented in Fig 1. The study was approved by the Volcani Center Animal Care Committee (approval no. IL 427/13); it involved 15 Israeli Holstein (6 mature bulls, aged 6.0 ± 1.3 years and 9 young bulls aged, 2 ± 0.3 years) working bulls from SION–Israeli Company for Artificial Insemination and Breeding Ltd., which participates in the breeding program of the Israeli Cattle Breeders Association. The bulls were housed in individual stalls and individually fed the basal total mixed ration that is routinely fed at SION [68.4% (w/w) dry matter, 7.2% (w/w) protein, 36.2% (w/w) neutral detergent fiber, 20% (w/w) acid detergent fiber, 1.45 net energy Mcal/kg, and 3.5 g minerals/kg (NaCl, Ca and P)]. Bulls were divided into three experimental dietary groups (n = 5 bulls each); each treatment group included two mature and three young bulls, which were fed with fatty acid supplement (SILA, Venice, Italy): (1) saturated fatty acids (the control group)– 360 g/day per bull with encapsulated fat from vegetable oil consisting of 64.2% palmitic acid (C16:0) and 34.8% stearic acid (C18:0); (2) the flaxseed oil group– 450 g/day per bull with encapsulated fat from flaxseed oil providing 84.2 g/day of alpha-linolenic acid; (3) the fish oil group– 450 g/day of encapsulated fat from fish oil providing 8.7 g/day eicosapentaenoic acid and 6.5 g/day docosahexaenoic acid. The amounts of supplement were adjusted based on the percentage of fat and ash; the saturated fatty acids diet contained 99% fat, and the flaxseed oil and fish oil diets contained 80% and 19% ash, respectively. The supplements were individually delivered and hand-mixed with the offered total mixed ration. Since the duration of spermatogenesis in bulls is about 61 days [30], the feeding period lasted for 13 weeks (i.e., 91 days); thus, it affected both the earlier and the later developmental stages through spermatogenesis and thereafter, through testis storage.

### Ejaculate collection

Ejaculates were collected once a week (from the first collection of each week) from each bull, starting 1 week before initiation of the feeding trial, through the feeding period (13 weeks), and 2 weeks after the feeding trial ended. Bulls were mounted on a live teaser, and semen was collected into a disposable tube using a sterile heated (38°C) artificial vagina. Ejaculates were immediately transferred to the laboratory and subjected to the cryopreservation procedure routinely conducted at SION [31]. Briefly, collected ejaculates were diluted (1:10 v/v at room temperature) with extender [10% (v/v) glycerol, 20% (w/v) egg yolk, 20 mg lactose, 1000 IU penicillin, and 500 mg streptomycin]. Then the samples were chilled for 3 h to 4°C and inserted into 0.25-mL chilled straws. Straws were cooled for 10 min to -95°C in a programmed box with a vapor nitrogen-saturated atmosphere, and then plunged into liquid nitrogen.

### In-vitro analysis

The analysis was conducted on samples that were collected 1 week before (-1 week), during (weeks 3, 7, and 11), and 2 weeks after (+2 weeks) the feeding trial (Fig 1A). The selected time points for analysis were based on our previous study [8] in which alterations in the fatty acid composition were first detected on week 7 of feeding and lasted until week 13, 2 weeks after the feeding trial ended.

The first examination included cell-based assessments of frozen–thawed samples: viability, mitochondrial membrane potential, reactive oxygen species (ROS) level, damaged acrosomal membrane, membrane fluidity, phosphatidylserine translocation, and the DNA fragmentation index (Fig 1B). All evaluations were conducted using a flow cytometer with specific fluorescent probes. Samples from each individual bull were analyzed; therefore, each bull represented one replicate within an experimental group (n = 15; 5 replicates per group). In addition, pooled samples, consisting of 5 straws/group, were examined for the maintained parameters. For each group, data recorded from the pooled samples were compared to a calculated average, i.e., an average of five bulls within each group for each of the examined parameters. Based on this comparison and due to a technical problem, DNA fragmentation and fertilization competence were conducted using pooled samples of two time points: before the feeding trial (-1 week) and +2 weeks after the trial ended. For each experimental group (i.e., saturated fatty acids, fish, or flaxseed oil), the pool consisted of one straw from each bull (n = 5).

Another set of examinations was performed to evaluate the *in-vitro* fertilization spermatozoa capacity and the developmental competence following fertilization (Fig 1C). Cumulus oocyte complexes (COCs) were *in-vitro* matured and randomly divided into three groups, and fertilized with the pooled spermatozoa (~1x10^6^), described above. Spermatozoa were collected 1 week before the feeding trial started (-1 week). The experiment included 3 IVF runs and 515 COCs in total.

Another set of COCs were *in-vitro* matured, randomly assigned into three groups, and *in-vitro* fertilized with the pooled spermatozoa (~1x106), as described above. Spermatozoa were collected 2 weeks after the feeding trial ended (+2 weeks). The experiment included 3 IVF runs and 216 COCs in total. Following fertilization, putative zygotes were cultured *in vitro* for 8 days. The fertilization rates were determined based on the proportion of oocytes that cleaved to the 2- to 4-cell-stage embryos at 42–44 h post fertilization. Embryo developmental competence was determined by the proportion of embryos that developed to the blastocyst stage on day 8 post fertilization. The kinetics of embryo development was recorded using a time-lapse system with an incubator, which enables continuous monitoring of embryo kinetics and development, as described in detail below. Blastocysts were individually collected from each experimental group (n = 12; 4 replicates) and subjected to mRNA extraction followed by RT-qPCR.

### Cell-based assessment of spermatozoa

#### Spermatozoa evaluation by flow cytometry

For each bull (Fig 1), two straws from five collection time points were thawed and examined for viability following cryopreservation. Then, the samples were subjected to the swim-up procedure to ensure that further analysis will be performed on viable motile spermatozoa [32]. Samples were washed once in prewarmed (38.5°C) NKM buffer (pH 7.4; 110 mM NaCl, 5 mM KCl, and 20 nM MOPS [3-N-morpholino propanesulfonic acid]) by centrifugation at 600x*g* for 10 min at room temperature, followed by 20 min at 38.5°C, allowing the viable motile sperm to swim up.

Sperm was evaluated using the Guava EasyCyte microcapillary flow cytometer with CytoSoft software (Guava Technologies, Inc., Hayward, CA, USA) and ready-to-use flow cytometry kits containing lyophilized fluorochromes in each well (IMV Technologies, L’Aigle, France) as previously reported [33]. Flow cytometry tests were performed on frozen samples using the Guava EasyCyte microcapillary flow cytometer with CytoSoft software (Guava Technologies; distributed by IMV Technologies). The device detects particle-emission properties with three photomultiplier tubes (green: 525/30 nm, yellow: 583/26 nm, and red: 655/50 nm) and accommodated optical filters and splitters. Assessment included plasma membrane integrity, mitochondrial membrane potential, acrosomal membrane integrity, and ROS level. In addition, the DNA fragmentation index, the phosphatidylserine translocation, and membrane fluidity were examined by flow cytometry using specific probes [acridine orange, merocyanine 540, and annexin V (AV), respectively]. A signal from 5000 spermatozoa was counted for each sample within each examined parameter.

Calibration was performed using the EasyCyte Check Kit (ref. 023066; IMV Technologies) according to the manufacturer’s instructions. Briefly, 10 ΔL of EasyCheck reagent beads was diluted in 190 ΔL of EasyCheck diluent, mixed thoroughly, and run through the instrument in three replicates. The obtained results were compared to the intensity information attached to the kit for green and red lasers. Only runs with adjusted values for all lasers were used for further analysis.

#### Plasma membrane integrity

**Fig 2:**
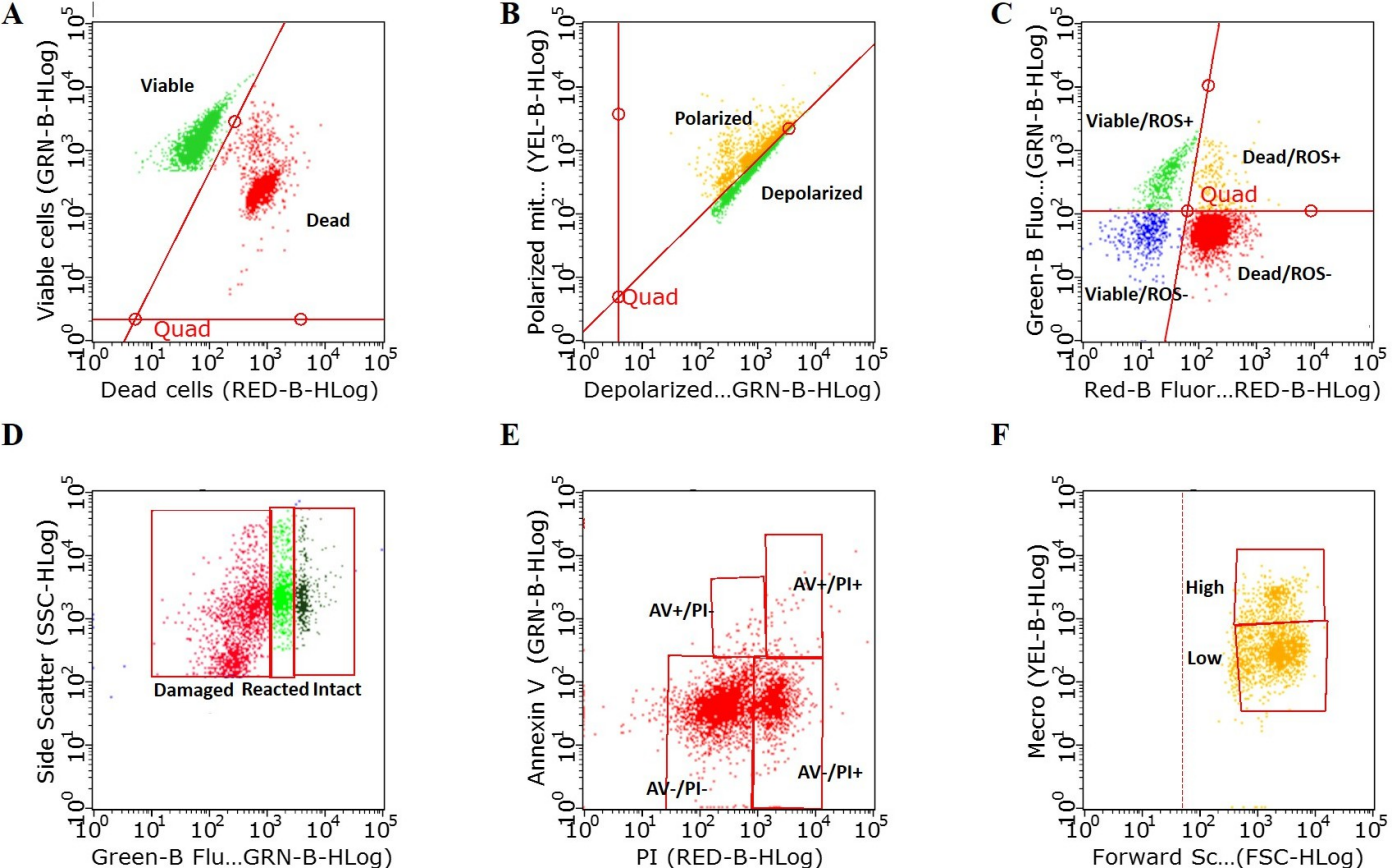
Evaluation of spermatozoa using the Guava EasyCyte microcapillary flow cytometer. Scatter plots of (A) viable and dead spermatozoa, (B) polarized and depolarized spermatozoa, (C) viable spermatozoa with or without ROS and dead spermatozoa with or without ROS, (D) spermatozoa with damaged, reacted, and intact acrosomal membranes, (E) annexin V (AV) positive (+) with negative (-) and positive (+) propidium iodide (PI), and AV- with PI- and PI+ spermatozoa, and (F) high- and low-intensity merocyanine 540 staining of spermatozoa.

Plasma membrane integrity was evaluated using the EasyKit 1 Viability and Concentration (ref. 024708; IMV Technologies) according to the manufacturer’s instructions and as previously reported [33]. The kit is based on two fluorescent probes: SYBR and propidium iodide (PI). SYBR stains nucleic acid and labels all spermatozoon heads green, whereas PI penetrates only membrane-damaged spermatozoa and labels spermatozoon heads red (Fig 2A). From each sample, 2 ΔL of homogeneous spermatozoa at 57 x 10^6^/mL was added into the well of a 96-well plate containing 199 ΔL EasyBuffer B (ref. 023826; IMV Technologies). The contents of each well were homogenized by pipetting, and the plate was covered and placed in an oven at 38.5°C, and protected from light for 10 min. A total of 5000 spermatozoa were counted in the flow cytometry reading. The results are expressed as the percentage of viable spermatozoa.

#### Mitochondrial membrane potential

The mitochondrial membrane potential of the spermatozoa was evaluated using EasyKit 2 (ref. 024864; IMV Technologies) according to the manufacturer’s instructions and as previously reported [33]. The kit is based on the 5,5’,6,6’-tetra-chloro-1,1’,3,3’-tetraethylbenzimidazolyl carbocyanine iodide (JC-1) probe, which changes color based on the mitochondrial membrane potential (Fig 2B). A volume of 10 ΔL absolute ethanol was added into each well to suspend the fluorochrome, followed by the addition of 190 ΔL phosphate buffered saline (PBS). Then, 2 ΔL of homogeneous spermatozoa at 57 x 10^6^/mL from each sample was added separately into the wells of a 96-well plate, homogenized by pipetting, and the plate was covered and placed in an oven at 38.5°C, and protected from light for 30 min. A total of 5000 spermatozoa were counted. The results are expressed as the ratio between the percentages of spermatozoa expressing polarized and depolarized mitochondrial membranes.

#### Reactive oxygen species level

The level of ROS was evaluated with EasyKit 3 (ref. 025157; IMV Technologies) according to the manufacturer’s instructions and as previously described by Sellem et al. [34]. The kit is based on the probe dihydrorhodamine 123 (DHR123), which evaluates the intracellular level of hypochlorous acids that are ROS (Fig 2C). A volume of 2 ΔL of homogeneous sperm at 57 x 10^6^/mL was added into each well of a 96-well plate containing 199 ΔL prewarmed PBS (38.5°C). The contents of each well were homogenized by pipetting, and the plate was covered and placed in an oven at 38.5°C, and protected from light for 20 min. Then, 2 ΔL of 39 mM hydrogen peroxide was added into each well, and incubated for an additional 40 min in an oven at 38.5°C, and protected from light. Thereafter, spermatozoa were washed with 600 ΔL prewarmed PBS at 38.5°C and centrifuged for 5 min at 300 x g. The pellet was suspended in 200 ΔL PBS, placed in a 96-well plate, and loaded into the flow cytometer. A signal from 5000 spermatozoa was counted, and the results were expressed as the distribution of ROS+ viable spermatozoa relative to ROS- spermatozoa.

#### Acrosomal membrane integrity

The acrosomal membrane integrity was evaluated with EasyKit 5 (ref. 025293; IMV Technologies) according to the manufacturer’s instructions with minor modifications, as previously described [33]. The kit is based on fluorescein isothiocyanate-labeled peanut agglutinin (FITC-PNA) that binds to the inner surface of the outer acrosomal membrane, which is accessible after an acrosome reaction, i.e., after the membrane has been ruptured [35]. According to the FITC-PNA labeling, a damaged acrosomal membrane expresses a positive green fluorescence signal, whereas an intact acrosomal membrane does not (Fig 2D). A volume of 2 ΔL of homogeneous spermatozoa at 57 x 10^6^/mL was added to each well of a 96-well plate containing 199 ΔL EasyBuffer B (ref. 023826; IMV Technologies) (38.5°C). The contents of each well were homogenized by pipetting, and the plate was covered and placed in an oven at 38.5°C, and protected from light for 45 min. Then, the plate was loaded into the flow cytometer for signal reading. A signal from 5000 spermatozoa was counted, and the results were expressed as the percentage spermatozoa with a damaged acrosomal membrane.

#### Phosphatidylserine translocation and membrane fluidity

Phosphatidylserine translocation and sperm membrane fluidity were evaluated by combined staining with AV–FITC dye (MACS Miltenyi Biotec GmbH, Bergisch Gladbach, Germany), PI, and merocyanine 540. Prior to the analysis, fluorescence compensation was conducted to correct the overlap between the emission spectra of PI and merocyanine 540, thus ensuring that the detected and measured signal derives from the fluorochrome. A sample of 57 x 10^6^/mL spermatozoa (~ 5 ΔL) was added to a tube containing 3 ΔL AV–FITC dye and 30 ΔL of binding buffer (MACS Miltenyi Biotec GmbH) diluted (1:20) with ultrapure water. The tube was incubated for 15 min in the dark at room temperature; then the contents were washed with 1 mL binding buffer, centrifuged, and 200 ΔL of diluted (1:20) binding buffer was added to the supernatant, mixed, and transferred to the well of a 96-well plate. A volume of 1 ΔL of 540 ΔM merocyanine 540 was added into the well and the plate was incubated for 10 min, and protected from light at room temperature. Immediately before reading, 0.3 ΔL PI was added to each well. The use of three dyes enabled distinguishing between three categories: (1) no changes in phosphatidylserine translocation corresponding to a non-apoptotic event (a negative fluorescent signal of both AV and PI), (2) phosphatidylserine translocation corresponding to an early apoptotic event (a positive AV fluorescent signal combined with a negative PI signal), and (3) phosphatidylserine translocation combined with membrane damage, corresponding to a late apoptotic event (a positive fluorescent signal for both AV and PI) as described by Shen et al. [36] (Fig 2E). In addition, the spermatozoa characterized as non-apoptotic were classified into high- and low-fluidity membranes corresponding to a high and low fluorescent signal of merocyanine, respectively (Fig 2F).

#### DNA fragmentation index

The spermatozoa DNA fragmentation index was evaluated by chromatin assay using acridine orange dye and a flow cytometer reader as described previously [37]. A sample containing 1 x 10^6^ spermatozoa was diluted in 147 ΔL Tris/NaCl/EDTA buffer (0.01 M Tris-HCl, 0.15 M NaCl, 1 mM EDTA, and pH 7.4) and incubated with 300 ΔL detergent acid solution (0.17% w/v Triton X-100, 0.15 NaCl, 0.08 N HCl, and pH 1.2) in a 1.7-mL microcentrifuge tube for 30 s at room temperature. Then, 900 ΔL of acridine orange solution at a final concentration of 6 Δg/mL (0.15 M NaCl, 1 mM EDTA, 0.1 M citric acid, 0.2 M Na_2_HPO_4_, and pH 6.0) was added. The tube was loaded into the flow cytometer and read for 2.5 min. The DNA-compaction level was expressed as the DNA fragmentation index, i.e., the percentage of spermatozoa with fragmented DNA.

### Fertilization competence and embryonic development

#### In-vitro production of embryos

*In-vitro* production of bovine embryos was performed as previously described [28, 29, 37]. Bovine ovaries were collected at a local slaughterhouse from multiparous Holstein cows, and transported to the laboratory. COCs were aspirated and subjected to *in-vitro* maturation, i.e., incubated for 22 h in humidified air with 5% CO_2_ for 22 h at 38.5°C. At the end of maturation, *in-vitro* fertilization was conducted with pooled samples (n = 5 straws, one from each bull/experimental group) collected 1 week before and +2 weeks after the feeding period. *In-vitro* culture was performed in a conventional incubator or in a TLS with an incubator (Miri; ESCO Medical, Egaa, Denmark). For the conventional incubator, putative zygotes were placed in groups of 10 in a 25-μL potassium simplex optimized medium (KSOM) droplet covered with mineral oil. For the TLS incubator, putative individual zygotes were placed on a CultureCoin (ESCO Medical) culture slide. Each of the 14 microwells of the CultureCoin were filled with 25 μL KSOM, covered with mineral oil, and inserted into the TLS incubator, which enables continuous monitoring of embryo kinetics and development. In both incubators, embryos were cultured for 8 days at 38.5°C in a humidified atmosphere of 5% CO_2_, 5% O_2._

Embryo kinetics and development were recorded for up to 190 h (8 days) post fertilization. Automatic time-lapse imaging was programmed to take images of each individual embryo every 5 min throughout the culture period. The images were taken through seven focal planes by a built-in Zeiss objective (20×) with a numerical aperture of 0.35 designed for 635 nm illumination using red light. The individual time-lapse images were then assembled into AVI movies by Miri® TL software. The precise timing of the 1^st^, 2^nd^, and 3^rd^ divisions (corresponding to 2-, 4-, and 8-cell-stage embryos, respectively) and blastocyst formation was annotated and expressed in hours post fertilization. The time of fertilization, i.e., the time when spermatozoa were added to the culture, was defined as time zero (t = 0). The cleavage rate into 2- to- 4-cell-stage embryos and the blastocyst formation rate were evaluated 42 h and after ~190 h post fertilization, respectively.

#### Blastocyst transcript abundance

Gene expression was analyzed on single in-vitro-derived blastocysts developed following fertilization with pooled samples (collected at +2 weeks after the end of the feeding trial). Blastocysts were collected on day 8 post fertilization and stored at -80°C until mRNA extraction. Poly(A) RNA was isolated using the Dynabeads mRNA DIRECT Kit according to the manufacturer’s instructions (Life Technologies, Carlsbad, CA, USA) as previously described [28]. Briefly, samples were lysed and mixed with prewashed Oligo (dT)_25_ Dynabeads. Following mRNA binding, each sample was washed twice with buffer A, twice with buffer B, and finally, mRNA was eluted with 10 mM Tris–HCl. The purified mRNA was used as the template in cDNA synthesis with SuperScript® III Reverse Transcriptase (Life Technologies).

The qRT-PCR assay was carried out with primers for some marker genes that are essential for proper development of the preimplantation embryo. These included the Interferon (IFN)-stimulated gene 15 (*ISG15*), which is involved in the development of early bovine embryos and regulates IFNƮ expression in the developing blastocyst [38]); prostaglandin-endoperoxide synthase 2 (*PTGS2*), which is expressed in the embryo trophectoderm and plays a role in implantation [39]; Actin Alpha 2 (*ACTA2*), a molecular marker, which in bovine, is associated with embryo developmental competence [40]; Placenta Associated 8 (*PLAC8*), which is involved in placental development [41]; Signal transducer and activator of transcription 3 (*STAT3*), a quality marker for bovine gametes [42] and is involved in inner cell mass development in bovine blastocysts [43]; Sex determining region Y-box 2 (*SOX2*), a marker for bovine inner cell mass, which is involved in the developmental capacity regulation of the embryo [44]; POU domain, class 5 transcription factor 1 (*OCT4*), *which* is expressed at early developmental stages and is involved in the differentiation of the inner cell mass and the trophectoderm layers [45]; DNA methyltransferase 1 (DNMT1) plays a role in the maintenance of hemi-methylated strands during DNA replication [46] and has an essential role during bovine preimplantation development [47]; DNA methyltransferase 3B (*DNMT3B*) is essential for de novo methylation [48]; Tyrosine 3-monooxygenase/tryptophan 5-monooxygenase activation protein zeta (*YWHAZ*) and succinate dehydrogenase complex flavoprotein subunit A (*SDHA*) served as internal reference genes [28].

The primers were derived from bovine sequences found in Genbank and specific primer pairs were designed using Primer 3.0 software (Table 1). RT-qPCR was conducted using the LightCycler® 96 system (Roche, Basel, Switzerland) with the SYBR® Green qPCRBIO SyGreen Blue Mix Hi-ROX Kit (PCR Biosystems Ltd., London, UK) in a final volume of 20 μL containing ultrapure water (Biological Industries), 400 nM of each primer, and 3 μL diluted cDNA (1:4, v/v). A negative control, without reverse transcriptase, was included to ensure the absence of DNA template contamination. The reaction efficiency ranged between 90 and 110% with R2 > 0.995. The amplification program included preincubation at 95°C for 10 s to activate taq polymerase, followed by 40 amplification cycles of denaturation at 95°C for 10 s and annealing–elongation at 60°C for 15 s. All samples were run in duplicate in 96-well plates. Melting-curve analysis was performed at the end of the amplification to confirm single-gene specificity. Fluorescence was recorded to determine the threshold cycle during the log-linear phase of the reaction in which fluorescence rises above the background. Gene expression was quantified and analyzed by LightCycler® 96 software ver. 1.1 and the ΔΔC_t_ method was used to calculate the relative expression of each gene (i.e., the fold change). Normalization was conducted using the geometric mean of two internal reference genes, *YWHAZ* and *SDHA*, and against the control group. The stability of the chosen reference genes was previously assessed in blastocysts [28, 29, 37, 49], demonstrating stable expression at this embryonic developmental stage and an independent manner of the experimental group.

**Table 1:** Primers used for qRT-PCR analysis.

Gene	Primer	Accession number	Sequence (5’→3’)	Size (bp)
*ISG15*	Forward	NM_174366	CTGCTGGTGGTGCAGAACT	84
	Reverse		CTGCTTCAGCTGGACCTCAT	
*PTGS2*	Forward	NM_174445	GAAATGATCTACCCGCCTCA	161
	Reverse		TCTGGAACAACTGCTCATCG	
*ACTA2*	Forward	NM_001034502	CGAGGCTATTCCTTCGTGAC	103
	Reverse		CAGTGGCCATCTCATTCTCA	
*PLAC8*	Forward	NM_001025325	GGCAGACTGGCATCTTTGAC	116
	Reverse		CCATAGGCAGCATTCATTCA	
*STAT3*	Forward	NM_001012671	GTCGGCTACAGCCATCTTTGT	118
	Reverse		CCTGTCAACCCGTTTGTCTT	
*SOX2*	Forward	NM_001040483	GTCCTATGGTGCTGGATGCT	113
	Reverse		GTTGATGTTCATGGCACAGG	
*OCT4*	Forward	NM_174580	GTGAGAGGCAACCTGGAGAG	109
	Reverse		ACACTCGGACCACGTCTTTC	
*DNMT1*	Forward	NM_182651	GCTTTACTGGAGCGATGAGG	103
	Reverse		GAAGTCCTGGAGGCACTGAG	
*DNMT3B*	Forward	NM_ 181813	AGAACTGGGCATCAAAGTGG	172
	Reverse		GCTTCCACCAATCACCAAGT	
*YWHAZ*	Forward	NM_00174814	GCATCCCACAGACTATTTCC	124
	Reverse		GCAAAGACAATGACAGACCA	
*SDHA*	Forward	NM_174178	GGGAGGACTTCAAGGAGAGG	112
	Reverse		TCAACGTAGGAGAGCGTGTG	

### Statistical analysis

Data were analyzed by JMP-15 software (SAS Institute, Inc., 2004, Cary, NC, USA). Treatments were compared by repeated measures ANOVA with a pretreatment value of each variable serving as a covariate. Treatment x time (feeding x week) interaction was tested using the proportion of viable spermatozoa, the proportion of spermatozoa with damaged acrosome membrane, a mitochondrial membrane potential ratio, and a proportion of spermatozoa with phosphatidylserine translocation. The proportion of embryos cleaved to the 2- to 4-cell stage, the proportion of blastocyst formation, and the DNA fragmentation index were analyzed as variables by one-way ANOVA, followed by the Tukey–Kramer test. Data are presented as the mean ± SEM. An overall comparison of the oxidation status (the proportion of ROS+ and ROS- spermatozoa) and the membrane fluidity (viable spermatozoa with high and low fluidity) as well as the distribution for the flaxseed and fish oil groups relative to the saturated fatty acids group between weeks for incidence data was performed by chi-square, followed by Fisher’s exact test, and compared to the saturated fatty acids group. The qRT-PCR data were analyzed according to the 2^-ΔΔCT^ method, expressing the fold change of each selected gene within experimental groups. Data were normalized against the saturated fatty acids group (expression was set to 1). The fold-change data for each gene were subjected to one-way ANOVA, followed by the Tukey–Kramer test. Data are presented as the mean ± SEM. For all analyses, *P* < 0.05 was considered significant; *P*-values between 0.05 and 0.1 were also reported as trends that might be real and noteworthy. Note, a minimal data set that include the values beyond the means and values used to build graphs is provided as a Supporting Information file.

## Results

### Effect of flaxseed and fish oil on cell-based assessment of spermatozoa

#### Spermatozoa viability

**Fig 3:**
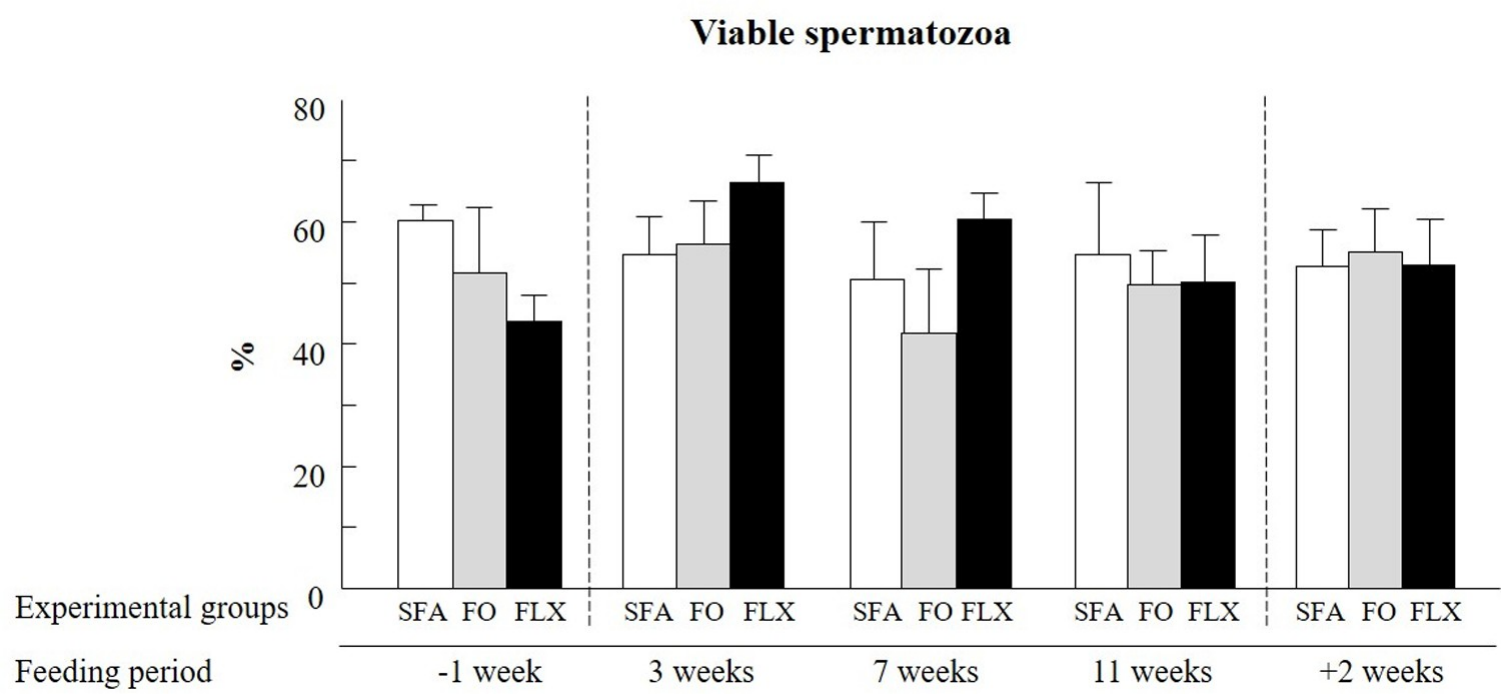
Effect of omega-3 sources flaxseed and fish oil on spermatozoa viability. The proportion of spermatozoa classified as viable, i.e., negative for propidium iodide (PI), as examined by flow cytometer. Experimental groups were fed saturated fatty acid (SFA; control), fish oil (FO), or flaxseed oil (FLX) supplements. Ejaculates were collected before (-1 week), during (3, 7, and 11 weeks), and after (+2 weeks) the feeding trial. Data are presented as the mean ± SEM calculated for 5 replicates with 5000 spermatozoa for each replicate.

The proportion of viable spermatozoa was similar between experimental groups; it was about 30–40% for all groups throughout the entire experimental period when examined immediately after thawing. Following the swim-up procedure, considering the treatment x time interaction effect, the proportion of viable spermatozoa remained similar (~53% viable spermatozoa) between and within experimental groups throughout the entire experimental period (*P* = 0.5; Fig 3).

#### Mitochondrial features

**Fig 4:**
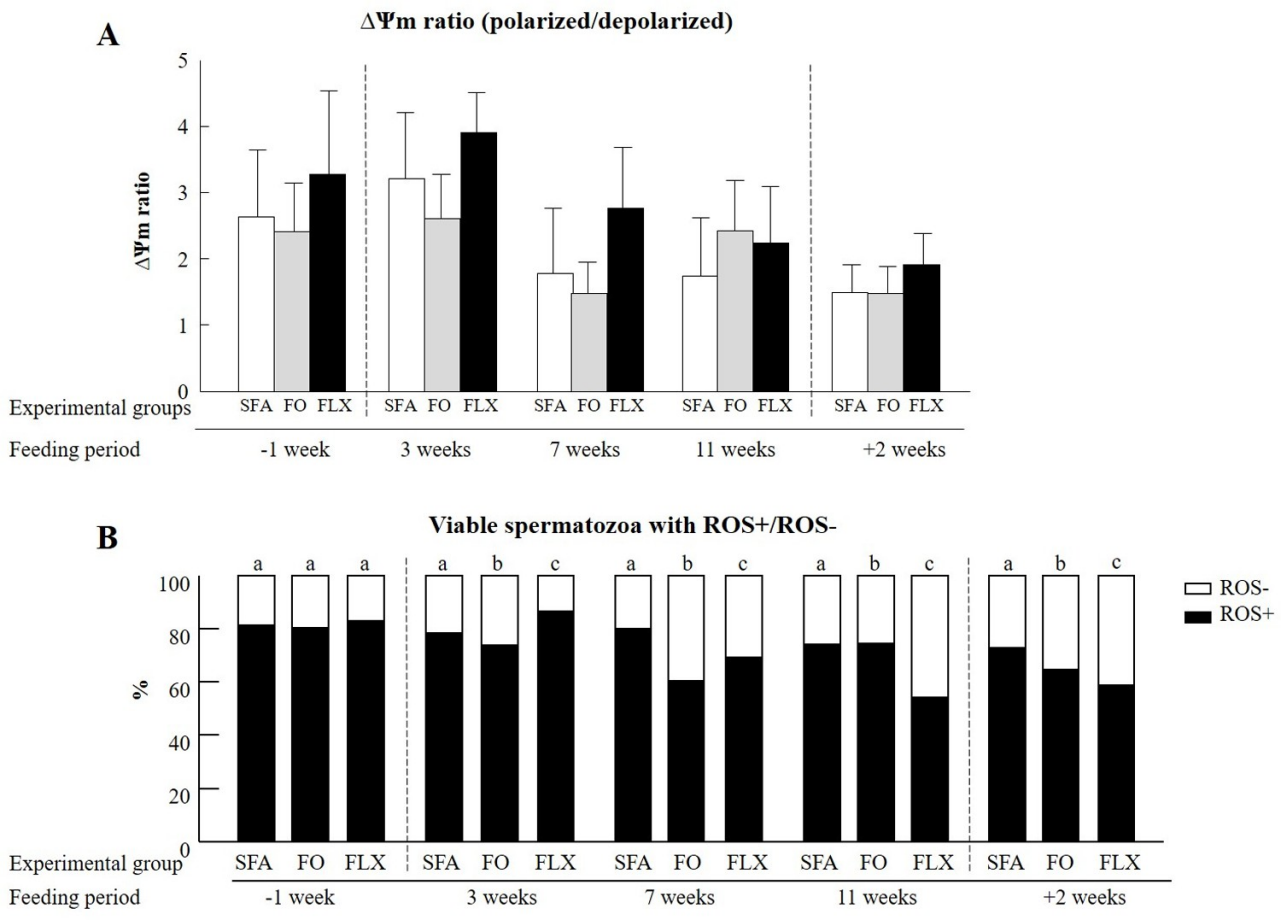
The effect of omega-3 sources flaxseed and fish oil on mitochondrial features. (A) The ratio of mitochondrial membrane potential (ΔΨm; polarized/depolarized) for spermatozoa from each experimental group: saturated fatty acid (SFA; control), fish oil (FO), and flaxseed oil (FLX), as determined by flow cytometer detection of the fluorescent probe JC-1 during the experimental period: before (-1 week), during (3, 7, and 11 weeks) and after (+2 weeks) the feeding trial. (B) The distribution of viable ROS+ and ROS- spermatozoa within each experimental group, as determined by flow cytometer detection of the fluorescent probe DHR123 during the experimental period. Data are presented as the mean ± SEM calculated for 5 replicates, 5000 spermatozoa for each replicate. Different letters above columns indicate significant differences between experimental groups at each time point (*P* < 0.05).

Neither flaxseed nor fish oil affected the mitochondrial membrane potential during or after the feeding period relative to the saturated fatty acids group, considering the treatment x time interaction effect (*P* = 0.99; Fig 4A). However, both flaxseed and fish oil affected the proportion of viable ROS+ spermatozoa (*P* < 0.0001; Fig 4B). For both the flaxseed and fish oil groups, the proportion of ROS+ viable spermatozoa was significantly reduced throughout the entire feeding period (*P* < 0.0001). In particular, feeding bulls with fish oil reduced the proportion of viable ROS+ spermatozoa relative to the saturated fatty acids group after 3 and 7 weeks of supplementation, and 2 weeks after the end of the feeding trial (*P* < 0.05; Fig 4B). Feeding with flaxseed oil resulted in a two-phase effect. After 3 weeks of supplementation, there was an increase in the proportion of viable ROS+ spermatozoa relative to the saturated fatty acids group (86.48 vs. 78.26%; *P* < 0.0001). From 7 weeks of flaxseed oil feeding up to 2 weeks after the end of the feeding trial, a decrease in the proportion of viable ROS+ spermatozoa was recorded (*P* < 0.0001; Fig 4B).

#### Acrosomal membrane integrity

**Fig 5:**
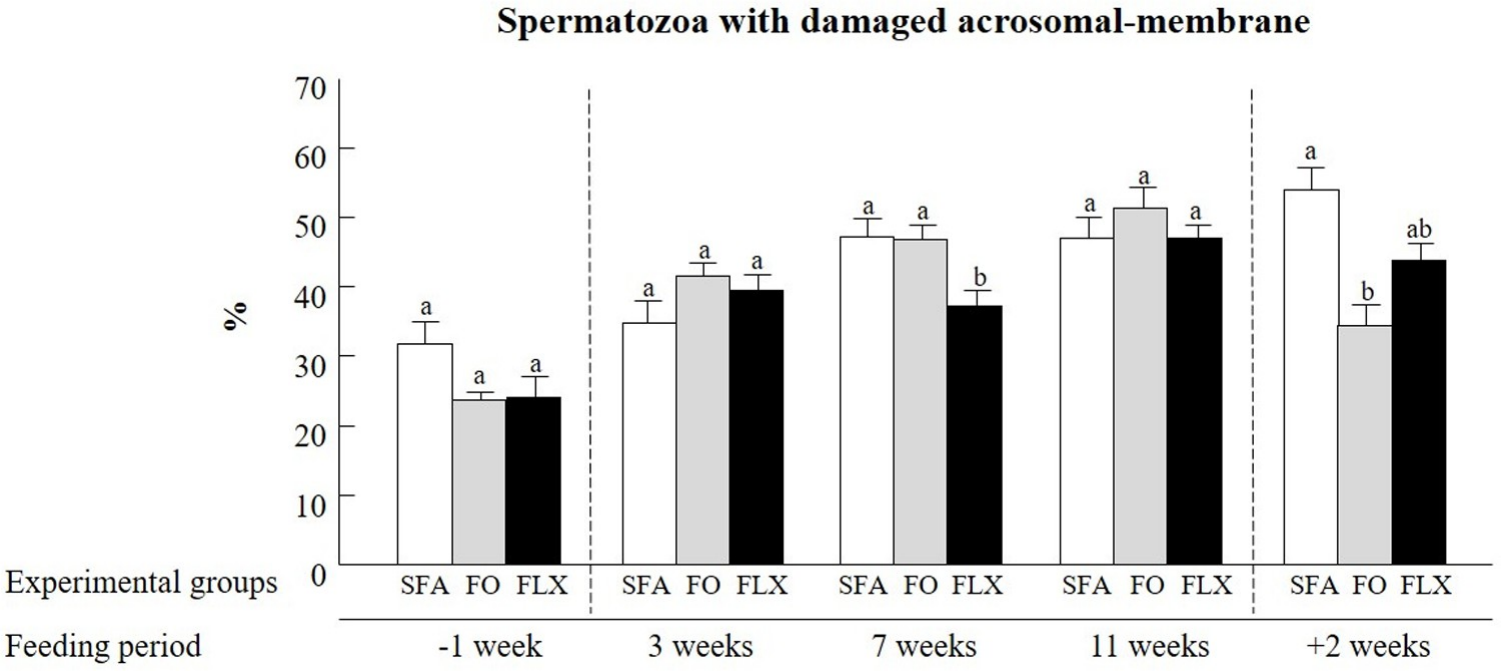
The effect of omega-3 sources flaxseed and fish oil on acrosomal membrane integrity. The proportions of spermatozoa exhibiting damaged acrosomal membrane, as determined by flow cytometer detection of the fluorescent probe FITC-PNA, are presented. The experimental groups were saturated fatty acid (SFA; control), fish oil (FO), and flaxseed oil (FLX). Ejaculates were collected before (-1 week), during (3, 7, and 11 weeks), and after (+2 weeks) the feeding trial. Data are presented as the mean ± SEM calculated for 5 replicates with 5000 spermatozoa for each replicate. Different letters above the columns indicate significant differences between experimental groups at each time point (*P* < 0.05).

Overall, treatment x time interaction indicated the significant effects of flaxseed and fish oil on acrosomal membrane integrity (*P* < 0.0001; Fig 5). Compared to the saturated fatty acids group, the proportion of spermatozoa with damaged acrosome membrane in the flaxseed oil group was lower at 7 weeks (*P* < 0.05), and tended to be lower 2 weeks after the end of the feeding trial (*P* < 0.06; Fig 5). Similarly, the proportion of spermatozoa with damaged acrosomal membrane was lower in the fish oil vs. the saturated fatty acids group 2 weeks after the end of the feeding trial (*P* < 0.05; Fig 5).

#### Phosphatidylserine translocation

**Fig 6:**
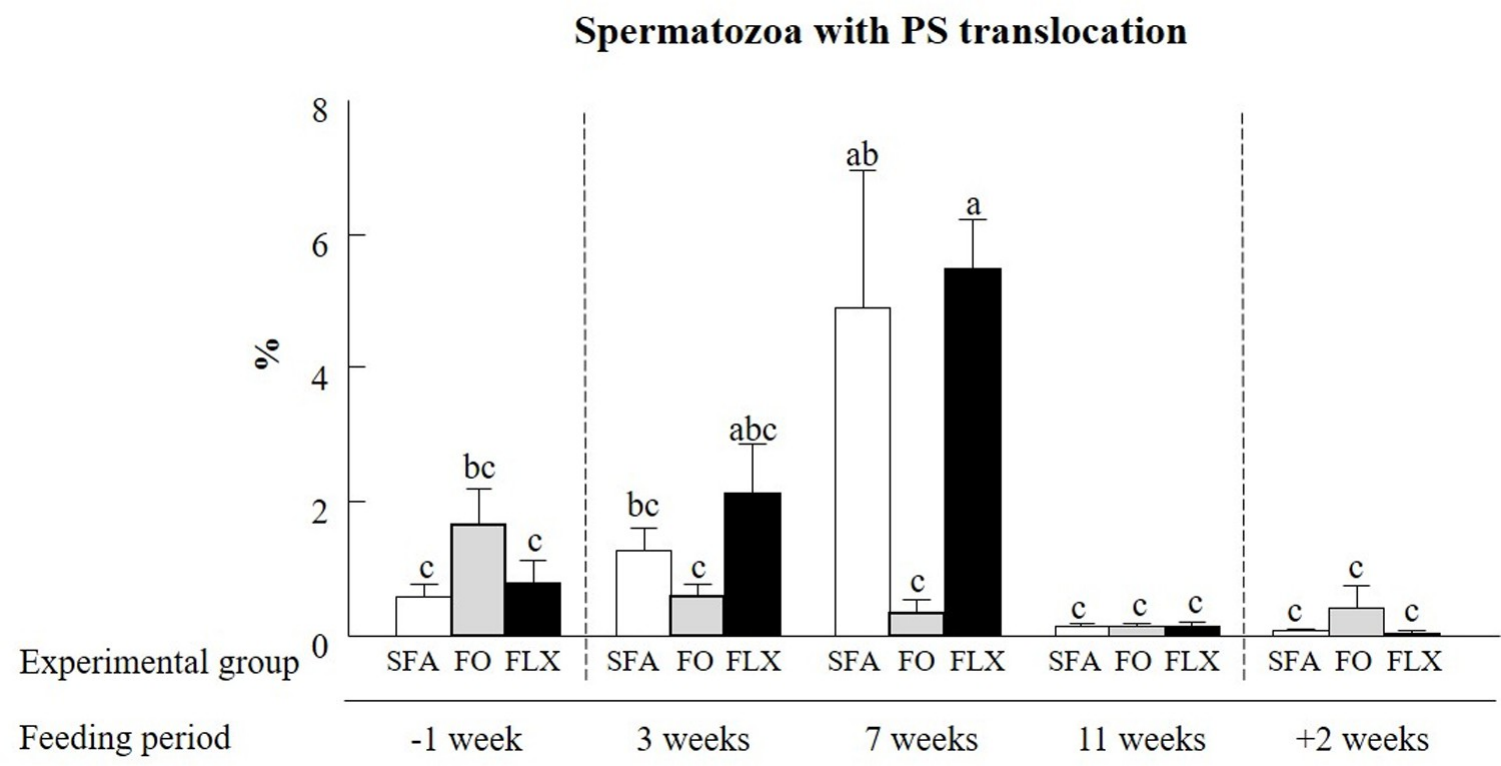
The effect of omega-3 sources flaxseed and fish oil on phosphatidylserine (PS) translocation. The proportion of spermatozoa with PS translocation within each experimental group: saturated fatty acid (SFA; control), fish oil (FO), and flaxseed oil (FLX), as determined by flow cytometer detection of the fluorescent probes annexin V and PI during the experimental period: before (-1 week), during (3, 7, and 11 weeks), and after (+2 weeks) the feeding trial is presented. Data are presented as the mean ± SEM calculated for 5 replicates with 5000 spermatozoa per replicate. Different letters above the columns indicate significant differences between experimental groups at each time point (*P* < 0.05).

Overall, treatment x time interaction indicated that feeding with flaxseed or fish oil only slightly affected the translocation of phosphatidylserine, corresponding to early apoptotic events (*P* < 0.05; Fig 6). In particular, a higher proportion of spermatozoa exhibiting phosphatidylserine translocation at 7 weeks after initiation of the feeding trial was found in the saturated fatty acids group relative to -1, 11, and +2 weeks of the feeding period (*P* < 0.05). Feeding with flaxseed oil resulted in the same pattern at 7 weeks, compared to -1, 11, and +2 weeks of the feeding trial (*P* < 0.05); it was similar to the saturated fatty acids group. However, feeding with fish oil resulted in a reduced proportion of spermatozoa exhibiting phosphatidylserine translocation events at 7 weeks after the initiation of the feeding trial relative to the saturated fatty acids and flaxseed oil groups (*P* < 0.05). There were no differences between the proportions of spermatozoa exhibiting phosphatidylserine translocation events at +2 weeks (Fig 6).

#### Membrane fluidity

**Fig 7:**
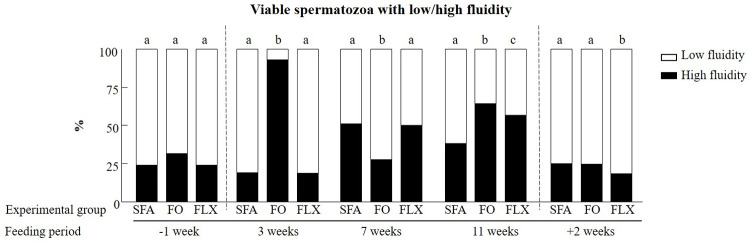
The effect of omega-3 sources flaxseed and fish oil on membrane fluidity in viable spermatozoa. The distribution of viable spermatozoa expressing high or low membrane fluidity within each experimental group: saturated fatty acid (SFA; control), fish oil (FO), and flaxseed oil (FLX) as determined by flow cytometer detection of the fluorescent probe merocyanine 540 during the experimental period: before (-1 week), during (3, 7, and 11 weeks), and after (+2 weeks) the feeding trial is presented. Different letters above the columns indicate significant differences relative to the SFA group at each time point (*P* < 0.05).

Feeding bulls with flaxseed or fish oil affected the spermatozoa membrane fluidity relative to the saturated fatty acids group (*P* < 0.0001; Fig 7). Before initiation of the feeding trial (-1 week), the ratio between high and low membrane fluidity was similar in all groups (*P* = 0.6; Fig 7). Feeding bulls with fish oil increased the proportion of viable spermatozoa with high membrane fluidity relative to the saturated fatty acids group at weeks 3 and 11 (*P* < 0.0001). At +2 weeks, the ratio between high and low membrane fluidity was similar in the fish oil and saturated fatty acids groups (*P* = 0.29; Fig 7). Feeding with flaxseed oil increased the proportion of viable spermatozoa exhibiting high membrane fluidity at 11 weeks relative to the saturated fatty acids group (*P* < 0.0001, Fig 7). However, this effect was no longer seen at +2 weeks (*P* < 0.0001; Fig 7).

#### DNA fragmentation index

Owing to technical limitations, the DNA fragmentation index was evaluated for pooled samples (5 bulls/group). Note that pooling was based on a preliminary comparison in which the calculated average data from individual bulls were compared with the data obtained from their pooled samples. No differences were found between the average and pooled data (Table 2); therefore, further examinations of DNA fragmentation were performed on the pooled samples.

**Table 2:** Comparison of the quality parameters from 5 bulls (i.e., the average data) with data of the pooled samples at -1 week and +2 weeks of the feeding period.

Experimental group	Week of the feeding period	Viable spermatozoa (%)	Spermatozoa with damaged acrosomal membrane (%)	Mitochondrial membrane potential (ratio)	Viable spermatozoa with ROS (%)
SFA-average	-1 week	60.14	31.86	2.64	81.19
SFA-pool		58.28	35.2	1.03	89.49
FO-average		51.61	23.81	2.41	80.42
FO-pool		51.56	27.04	2.44	83.77
FLX-average		43.61	24.16	3.28	83.07
FLX-pool		29.37	25.02	1.94	86.69
SFA-average	+2 weeks	52.59	54.04	1.49	72.79
SFA-pool		54.51	56.04	1.02	80.78
FO-average		54.99	34.46	1.47	64.77
FO-pool		51.02	36.06	2.54	65.24
FLX-average		52.88	43.75	1.91	58.6
FLX-pool		49.71	45.94	1.16	72.41

Comparisons were conducted between each experimental group within each feeding period separately.

SFA = saturated fatty acids; FO = fish oil; FLX = flaxseed oil.

Comparisons were performed on samples collected before (-1 week) and after (+2 weeks) the feeding period. No differences were found between the average and the pooled data (Table 2). In light of these findings, further examinations of DNA fragmentation were performed on the pooled samples.

**Fig 8:**
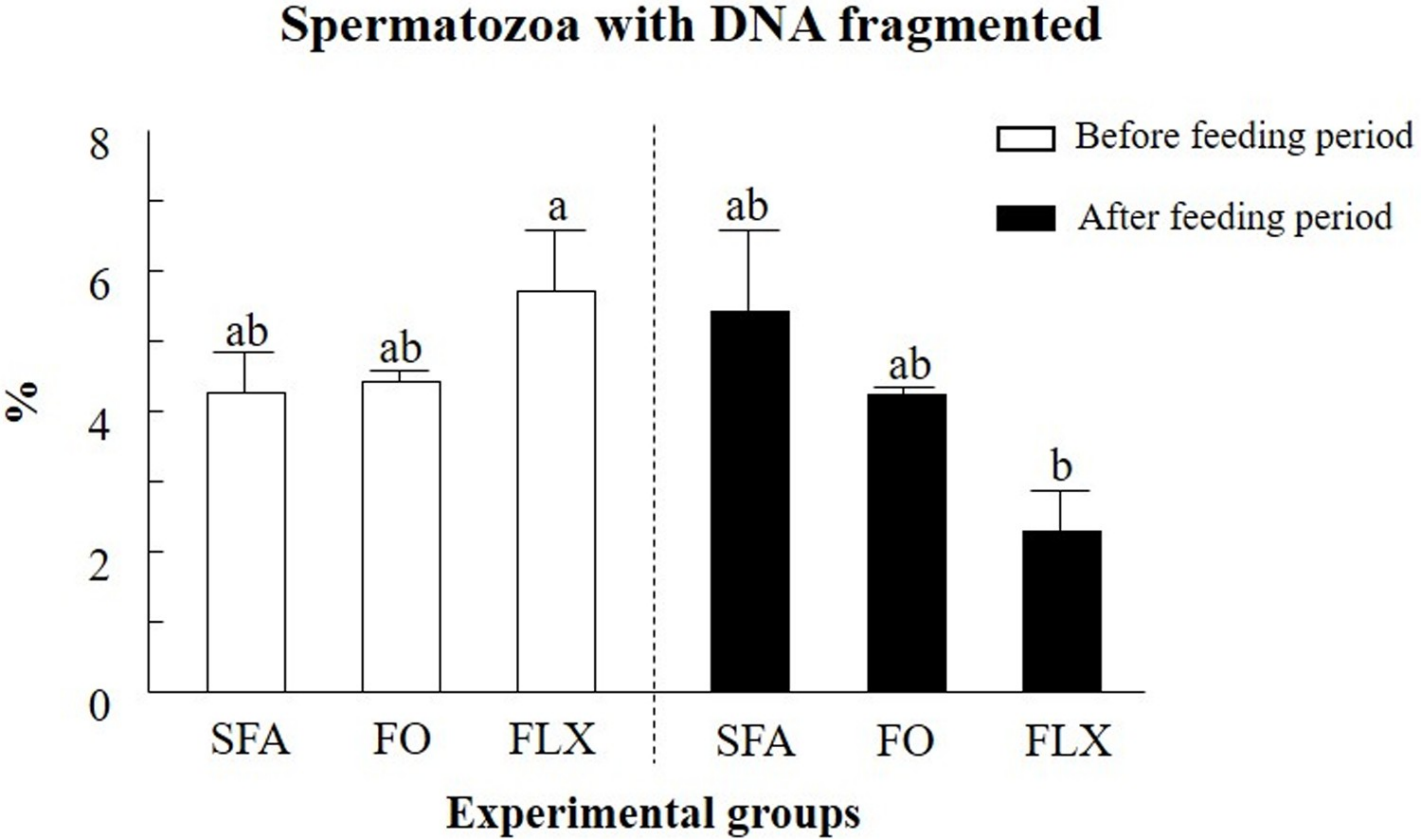
The effect of omega-3 sources flaxseed and fish oil on the DNA fragmentation index. The percentage of DNA fragmentation for pooled samples from each experimental group: saturated fatty acid (SFA; control), fish oil (FO) and flaxseed oil (FLX) taken from before and after the feeding trial is presented. Data are presented as the mean ± SEM. Different letters above the columns indicate significant differences relative to the SFA group at each time point (*P* < 0.05).

The DNA fragmentation index before the initiation of the feeding trial was similar between experimental groups. Feeding the bulls with flaxseed oil affected the DNA fragmentation index, as reflected by a significant decrease at +2 weeks relative to -1 week in the same experimental group (*P* < 0.05; Fig 8). In addition, a tendency toward a reduction in the DNA fragmentation index was recorded at +2 weeks in the flaxseed oil vs. the saturated fatty acids group (*P* < 0.06; Fig 8). Feeding with fish oil did not affect the DNA fragmentation index before or after the feeding trial.

### Effect of flaxseed and fish oil on Spermatozoa’s fertilization competence

Owing to the IVF limitations, the fertilization capacity was evaluated for pooled samples (5 bulls/group). Note that pooling was based on a preliminary comparison in which for each experimental group calculated average data from individual bulls were compared with the data obtained from their pooled samples. Comparisons were performed before (-1 week) and after (+2 weeks) the feeding period. No differences were found between the average and the pooled data (Table 2); therefore, further examinations of fertilization competence were performed on the pooled samples.

**Fig 9:**
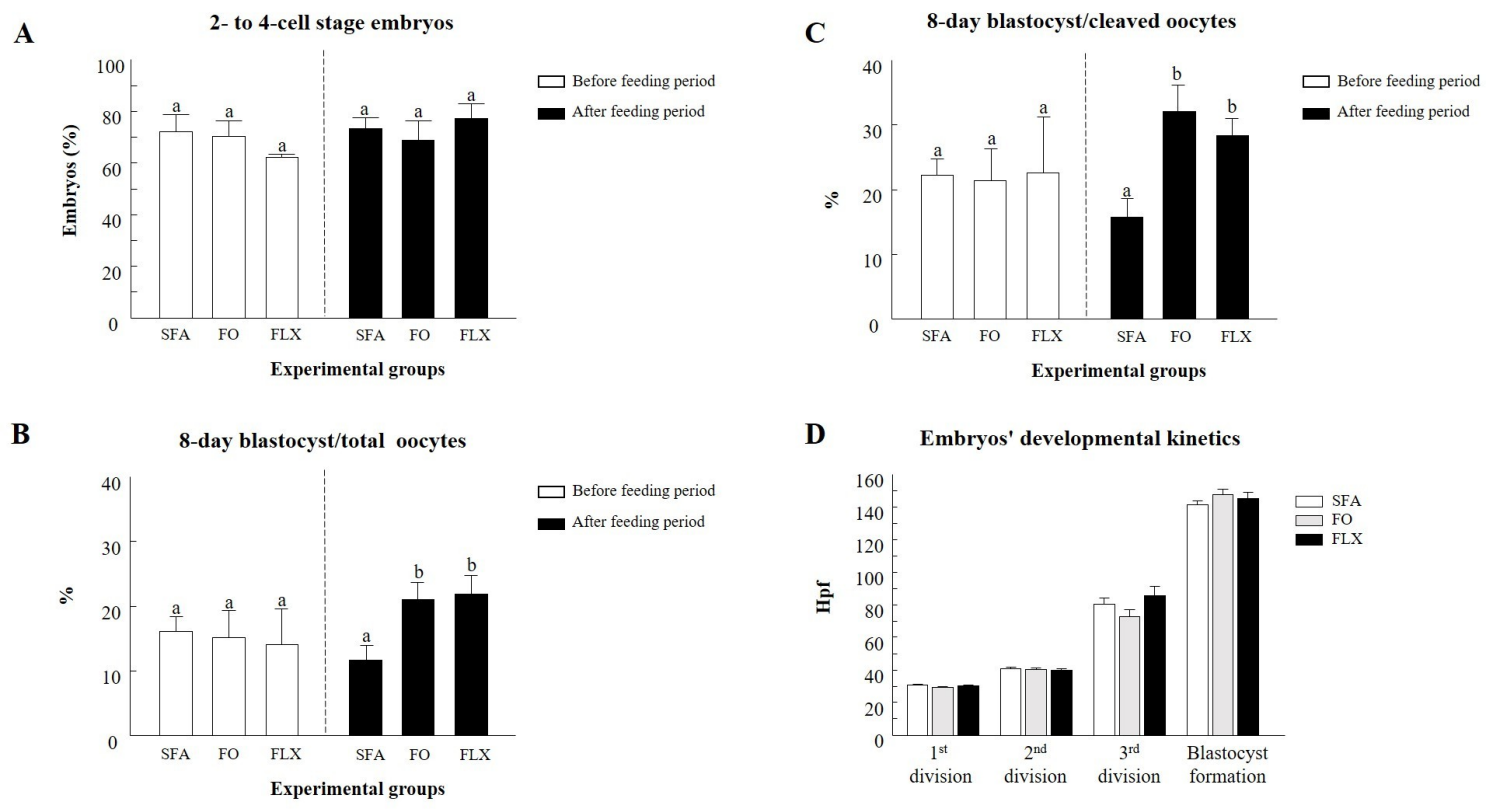
The effect of omega-3 sources flaxseed and fish oil on spermatozoa fertilization competence. (A) The proportion of 2- to 4-cell-stage embryos following *in-vitro* fertilization with pooled samples from each experimental group: saturated fatty acid (SFA; control), fish oil (FO), and flaxseed oil (FLX) taken from before and after the feeding trial. The proportion of embryos that developed into the blastocyst stage on day 8 post fertilization out of the total oocytes (B) or out of the cleaved oocytes (C). Data are presented as the mean ± SEM. (D) Embryo kinetics throughout the 1^st^, 2^nd^, and 3^rd^ divisions and blastocyst formation timing (hours post fertilization; hpf). Different letters above the columns indicate significant differences between groups (*P* < 0.05).

Fertilization with pooled samples collected before the initiation of the feeding trial resulted in a similar proportion of oocytes that cleaved into 2- to 4-cell-stage embryos, as well as those that developed into blastocysts (Fig 9A and 9B). Fertilization with samples collected after the feeding trial (+2 weeks) resulted in a significant increase in the proportion of blastocysts reported for both flaxseed and fish oil relative to the saturated fatty acids group (*P* < 0.05; Fig 9B). Further analysis, in which blastocyst formation was calculated relative to the proportion of cleaved embryos, revealed similar results (*P* < 0.05; Fig 9C). Embryo kinetics for the 1^st^, 2^nd^, and 3^rd^ divisions, as well as the timing of blastocyst formation, did not differ between experimental groups (Fig 9D).

#### Blastocyst transcript abundance

**Fig 10:**
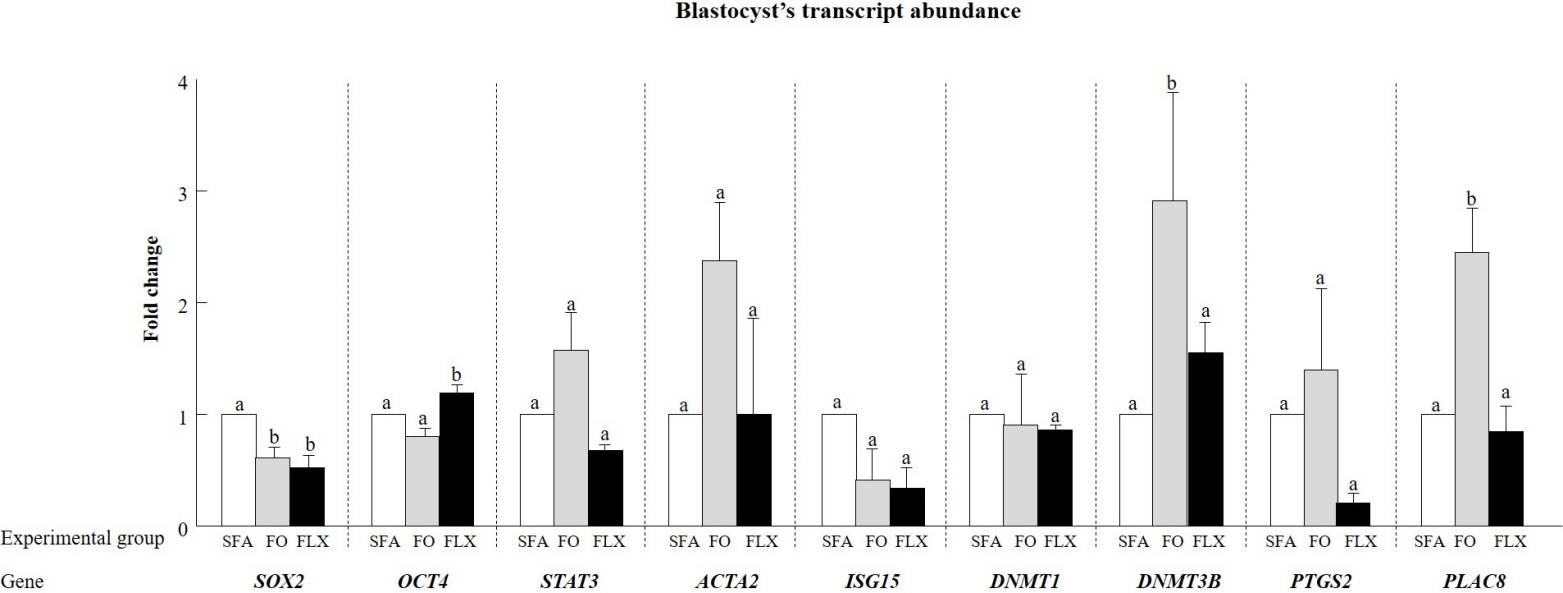
The effect of omega-3 sources flaxseed and fish oil on gene expression in embryos at the blastocyst stage. At 8 days post fertilization, the blastocysts developed from each experimental group: saturated fatty acid (SFA; control), fish oil (FO), and flaxseed oil (FLX), taken after the feeding trial, were collected, snap-frozen, and stored at -80°C for RNA extraction and qPCR analysis (n = 4 blastocysts per sample; 4 replicates). Transcript levels of *SOX2*, *OCT4*, *STAT3*, *ACTA2*, *ISG15*, *DNMT1*, *DNMT3B*, *PTGS2*, and *PLAC8* were normalized against the geometric mean of *YWHAZ* and *SDHA*. Data are expressed relative to the SFA group. Data are presented as the means ± SEM. Different letters above the columns indicate significant differences relative to the control group (SFA) for each gene (*P* < 0.05).

Gene expression was examined in blastocysts developed from pooled samples of saturated fatty acids, fish and flaxseed oil groups, collected 2 weeks after the feeding trial ended. The expression of *ACTA2*, *DNMT1*, and *PTGS2* did not differ between fish oil and saturated fatty acids blastocysts (Fig 10). The expression of *SOX2* was lower in fish oil vs. the saturated fatty acids blastocysts (*P* < 0.05), whereas the expression of *DNMT3B* and *PLAC8* was higher in fish oil vs. the saturated fatty acids blastocysts (*P* < 0.05). The expression of *OCT4* and *ISG15* tended to be lower in the fish oil vs. the saturated fatty acids blastocysts (*P* < 0.06 and *P* < 0.07, respectively). The expression of *STAT3* tended to be higher in the fish oil vs. the saturated fatty acids blastocysts (*P* < 0.1; Fig 10).

The expression of *STAT3*, *ACTA2*, *DNMT1*, *DNMT3B*, *PTGS2*, and *PLAC8* did not differ between the flaxseed oil and the saturated fatty acids blastocysts (Fig 10). However, the expression of *OCT4* was higher and that of *SOX2* was lower in the flaxseed oil vs. the saturated fatty acids blastocysts (*P* < 0.05; Fig 10). The expression of *ISG15* tended to be lower in the flaxseed oil vs. the saturated fatty acids blastocysts (*P* < 0.07; Fig 10).

## Discussion

The effect of omega-3 on spermatozoa quality within and across species is controversial. Although some studies report beneficial effects [1], others have found no effect [6, 50]. The current study strengthens some previous findings and explores new aspects regarding the effect of an omega-3 enriched diet on bovine cryopreserved spermatozoa. Flaxseed oil supplementation to the bull feed increased the proportion of spermatozoa with intact acrosome membrane and lowered DNA fragmentation, the proportion of ROS+ spermatozoa, and the proportion of those spermatozoa with high membrane fluidity post thawing. A beneficial effect was also found for bulls fed with fish oil, expressed by a lower proportion of ROS+ spermatozoa, and a reduced proportion of spermatozoa with damaged acrosome. Our findings suggest that omega-3 supplementation, of both plant and animal origin, can be used to enrich bull feed, thereby improving the male fertility and reproductive performance in dairy herds. In support of this assumption, in both treated groups, the previously mentioned changes were associated with a higher proportion of embryos that developed to blastocyst following *in-vitro* fertilization, relative to the control. In addition, the study provides the first evidence of the paternal effect, from the spermatozoa to the developed blastocyst, differential gene expression was recorded in embryos from the flaxseed and fish oil groups. In particular, prominent differences were recorded in genes involved in early embryonic development. Taken together, feeding bulls with an omega-3-enriched diet, regardless of the source, i.e., flaxseed or fish oil, meets dairy reproductive management needs, which are based on artificial insemination with cryopreserved spermatozoa. Note that spermatogenesis lasts ~61 days [30]; therefore, a long-lasting period of omega-3 feed enrichment is suggested.

### Effect of omega-3 on spermatozoa features

#### Membrane integrity

Integrity of the cell membrane is crucial for cell survival and serves to evaluate cell viability. In the current study, feeding bulls with flaxseed or fish oil did not have any beneficial effect on the proportion of viable spermatozoa during or after the feeding trial. Similarly, feeding boars with omega-3 for 26 weeks did not affect the spermatozoa viability in fresh semen [6], and feeding young bulls with fish oil for 12 weeks did not have any beneficial impact on the viability of spermatozoa post thawing [50]. In contrast, feeding Zandi rams with fish oil for 8 weeks increased the proportion of viable spermatozoa in freshly collected semen [14]. In addition, feeding buffalo bulls with flaxseed oil for 12 weeks improved the proportion of live spermatozoa, expressed from 8 to 12 weeks of treatment [51]. Similarly, feeding with flaxseed oil for 7 weeks enhanced spermatozoa viability in fresh samples from Friesian bulls [18]. Feeding Holstein bulls subjected to environmental heat stress with omega-3 for 12 weeks increased the proportion of viable spermatozoa at the end of the feeding trial using fresh semen, with no effect observed on frozen–thawed semen [9]. Alterations in the composition of the spermatozoa membrane via the process of cryopreservation [25–27] might at least partially explain the discrepancies between fresh and cryopreserved samples.

#### Mitochondrial features

The mitochondrion is the energy-producing apparatus, through the complex mechanism of oxidative phosphorylation [52], in which the mitochondrial membrane potential plays a pivotal role. Given the high correlation between mitochondrial membrane potential and spermatozoa motility, any changes in mitochondrial function might lead to physiological dysfunction, including male infertility [53]. In our previous study, feeding with flaxseed oil increased spermatozoa motility in fresh semen [8]. Therefore, we expected to find an increase in mitochondrial membrane potential. However, feeding with either flaxseed oil or fish oil had no beneficial effect on the mitochondrial membrane potential of spermatozoa examined post thawing. Similarly, feedings rams with fish oil for 16 weeks did not affect the mitochondrial membrane potential in frozen–thawed spermatozoa [54]. In humans, supplementation of docosahexaenoic acid (0.5, 1.0 or 2.0 g/day) for 1 and 3 months did not have any significant effect on spermatozoa mitochondrial membrane potential [55].

Although feeding with flaxseed or fish oil did not affect mitochondrial membrane potential, it positively affected the spermatozoa oxidation status, manifested by a lower proportion of viable ROS+, 3 weeks after the beginning of the feeding trial, throughout the entire feeding period, and 2 weeks after the feeding trial was terminated. These findings suggest that both treatments improve the spermatozoa oxidative status, i.e., the balance between the ROS level and antioxidant factors, such as glutathione peroxidase and superoxide dismutase [56]. Although moderate levels of ROS in spermatozoa are required for proper physiological function, including capacitation, acrosome reaction and interaction with the oocyte [57, 58], high ROS production, exceeding physiological levels, can induce oxidative stress, resulting in DNA and lipid damage [59]. In support of this, a significant correlation between omega-3 concentration and antioxidant activity was found in human seminal fluid [11]. In addition, a significant increase in antioxidant concentration was recorded in human seminal plasma following feeding with omega-3 supplement [60]. It is therefore suggested that enrichment with omega-3 sources (fish or flaxseed oil) enables maintaining the balance between ROS and antioxidant levels, most likely due to the increased proportion of PUFA. Although higher amounts of PUFA in the spermatozoa membrane can increase the risk of lipid peroxidation [22], a higher proportion of docosahexaenoic acid [8] can make this membrane more resilient to oxidative stress [61]. In support of this, culturing human aortic endothelial cells with 100 nM docosahexaenoic acid attenuated oxidative stress, expressed by a reduction in intracellular ROS levels [62]. In addition, fluctuation in ROS levels during the feeding trial, from 3 to 11 weeks, corresponded to alterations in the spermatozoa membrane fatty acid composition found 7 weeks into the feeding trial [8]. Similarly, feeding boars with cod liver oil for 9 weeks induced alterations in the semen fatty acid composition (i.e., increased docosahexaenoic acid) only at weeks 4–5 [63]. With spermatogenesis lasting ~61 days in bulls [30], these results imply that feeding bulls with fish or flaxseed oil for 13 weeks had an impact on the spermatozoa membrane fatty acid composition, presumably at an early phase of spermatogenesis. In support of this assumption, the effects on the ROS level, as well as on DNA fragmentation, membrane fluidity, phosphatidylserine translocation, and fertilization competence lasted for 2 weeks after the feeding trial ended, and these spermatozoa were most likely affected earlier.

#### Acrosome membrane integrity

Acrosome membrane integrity in mammalian spermatozoa, mainly in bulls and humans, is critical for the acrosome reaction. The acrosome membrane integrity and the acrosome reaction have been suggested to predict fertilization competence [64]. Thus, alterations in these features might result in reduced fertilization ability [65, 66]. Here we found that feeding bulls with flaxseed or fish oil had a beneficial effect, expressed by a lower proportion of spermatozoa with damaged acrosomal membrane relative to the saturated fatty acids group, 2 weeks after the feeding trail ended. In support of this, a beneficial impact of PUFA on acrosome-membrane integrity has been reported. Feeding young bulls with fish oil increased the proportion of spermatozoa with intact acrosome membrane at week 10 of feeding, and this effect lasted for up to 12 weeks [50]. In addition, feeding rams with fish oil for 14 weeks increased the percentage of spermatozoa with a normal acrosome membrane in fresh semen [15]. In another study, feed supplementation with flaxseed oil had several beneficial outcomes for Mithun (*Bos frontalis*) bulls, including a high proportion of spermatozoa with intact acrosome, in both fresh and frozen semen [67]. In agreement with this, lack of omega-3 in the diet of fatty acid desaturase-2 knockout mice affected the spermatid maturation and acrosome formation [68]. On the other hand, feeding young bulls with PUFA increased the proportion of frozen–thawed spermatozoa with damaged acrosomal membrane [19]. Therefore, the effect of omega-3 supplementation on acrosome membranes remains an open question.

#### Membrane fluidity

The proportion of PUFA in the spermatozoa membrane has been suggested to underlie its flexibility and fluidity [69, 70]. *In-vitro* culturing with docosahexaenoic acid was found to increase membrane fluidity in endothelial cells [71] and atherosclerotic-like model membranes [72]. In boar, dietary supplementation with omega-3 for 75 days increased spermatozoa membrane fluidity in fresh semen [73]. In buffalo, omega-3 supplementation (linseed oil) for 12 weeks increased spermatozoa membrane fluidity [74]. In our previous study, feeding bulls with flaxseed oil increased the proportion of α-linolenic acid, docosapentaenoic acid, and docosahexaenoic acid in the spermatozoa [8]. We therefore expected higher membrane fluidity within spermatozoa followed by treatment with flaxseed oil. The findings of the current study indicated a gradual increase in membrane fluidity from 7 to 11 weeks after feeding initiation with both fish and flaxseed oil. However, this effect did not last once the feeding treatments ended. Although spermatozoa expressed high levels of docosahexaenoic acid 2 weeks after the end of the feeding period, the proportion of spermatozoa with high membrane fluidity decreased to the level recorded before initiation of the feeding treatment. Although not entirely clear, Stillwell and Wassall [75] suggested only a weak association between membrane fluidity and docosahexaenoic acid content.

#### Membrane phosphatidylserine translocation

The phospholipids phosphatidylethanolamine, phosphatidylcholine, and phosphatidylserine contribute to membrane fluidity, fusion, fission, and flexibility [76]. Phosphatidylserine, located in the inner leaflet of the membrane [77], is likely to temporarily translocate from the inner to outer membrane [78] upon capacitation [79] or acrosome reaction [80]. In the present study, we utilized the AV assay; AV is a protein that binds selectively to phosphatidylserine [81]. The proportion of spermatozoa that were AV+ (i.e., cells with a scrambled membrane) was relatively low (2%) and did not differ between groups throughout the feeding period. Similar findings have been recorded in canines, for both fresh and frozen–thawed samples [82]. In the current study, during the feeding trial, an increase in the proportion of spermatozoa exhibiting phosphatidylserine translocation was observed at 7 weeks in the saturated fatty acids and flaxseed oil groups; however, this did not last beyond the feeding period, suggesting a short-term effect of feeding. The effect of omega-3-enriched diet on phosphatidylserine translocation thus remains unclear.

#### DNA fragmentation

DNA integrity, for instance, is considered a marker for fertility assessment [83]. Spermatozoa with intact DNA are associated with high fertilization competence and early embryonic development [84], whereas spermatozoa with fragmented DNA are associated with low fertility and a low blastocyst formation rate [85–88]. In the current study, feeding bulls with flaxseed oil had a positive impact on spermatozoa DNA fragmentation, which is associated with blastocyst formation, but not with the cleavage rate as discussed below. On the other hand, reduced DNA fragmentation in the flaxseed-treated group was associated with a reduced proportion of ROS+ spermatozoa. In humans, supplementation of docosahexaenoic acid reduces spermatozoa DNA fragmentation and increases the concentration of antioxidants in the seminal fluid [60], suggesting an improved oxidative status [89]. Previous studies conducted with infertile men demonstrated that combined treatment with antioxidants and essential fatty acids reduces the seminal ROS levels and the spermatozoa DNA damage [90]. Although the mechanism by which omega-3 protects spermatozoa DNA is not yet clear, a strong correlation between phosphatidylserine translocation and DNA damage has been reported in human [91] and bovine [37] spermatozoa.

### Effect of omega-3 on fertilization competence and blastocyst gene expression

#### Fertilization competence

Feeding bulls with flaxseed or fish oil improved the spermatozoa physiological parameters, such as a reduction in the ROS level and the DNA fragmentation index; however, this was not reflected in higher fertilization capacity, since the proportion of oocytes that fertilized and cleaved into 2- to 4-cell-stage embryos did not differ between groups. In contrast, feeding Zandi rams with fish oil increased the cleavage rate 48 h after *in-vitro* fertilization [92]. Nonetheless, feeding with flaxseed oil and fish oil had a beneficial impact on embryonic development, as discussed below.

#### Paternal effect on the developing blastocysts

Accumulating data emphasize the contribution of spermatozoa to the developing embryo. Here we report that feeding bulls with fish and flaxseed oil had a beneficial effect on embryonic development, expressed by a high proportion of *in-vitro*-derived blastocysts, suggesting a paternal effect on the formed embryo. Supporting this, a previous study on dairy cows indicated the beneficial impact of a flaxseed oil-enriched diet on the developing blastocyst, expressed by a high number of blastomeres in the developing embryos [93], and reduced incidence of pregnancy loss [94]. In contrast, Bastos et al. [19] did not find any effect on cleavage or the blastocyst-formation rate following PUFA supplementation to young bulls. The difference between findings might be related to differences in bull age or genotype, the source of supplement, or the duration of the feeding trial.

In the current study blastocysts that developed from fish and flaxseed oil spermatozoa exhibited a low expression of *SOX2*. In bovine, *SOX2* expression is highly associated with inner cell mass formation, but overexpression of *SOX2* at the blastocyst stage has a negative effect on the embryo’s developmental potential [44]. In addition, an increase in the expression of *OCT4* was recorded for blastocysts developed from flaxseed oil spermatozoa. Bovine blastocysts with higher expression of *OCT4* have better morphology, with a higher number of blastomeres [95]. It has been suggested that blastocysts with certain threshold levels of *OCT4* and *SOX2* expression are most likely to reach the elongation stage [95]. In addition, blastocysts developed from the fish oil group exhibited higher expression of *DNMT3B*, a gene required for *de-novo* methylation and for establishing new DNA-methylation marks [48]. Interestingly, in bovine, *DNMT3B* is differentially expressed between genders, with higher expression in male vs. female blastocysts [96]. It is therefore possible that feeding bulls with omega-3 might have some effect on the ratio between X and Y spermatozoa through spermatogenesis. This point requires further investigation. Blastocysts developed from the fish oil group exhibited higher expression of *PLAC8*, which plays a crucial role in embryo development [97]. In bovine blastocysts, *PLAC8* expression is suggested as a biomarker for pregnancy outcome [98]. Therefore, a higher expression of *PLAC8* might imply a higher chance for these embryos to hatch and implant.

## Conclusions

The present study supports the notion that an omega-3-enriched diet can affect not only fatty acid composition in the spermatozoa but also their quality, as expressed by decreased acrosome damage, ROS levels, and DNA fragmentation. Moreover, these effects are not limited to the feeding period; they are also expressed up to 2 weeks afterwards. A differential effect of omega-3 sources (flaxseed oil vs. fish oil) on the spermatozoa was further associated with embryo development and gene expression in the formed blastocysts, suggesting a carry-over effect. Overall, it can be generally implied that an omega-3-enriched diet, whether supplemented with flaxseed oil (a plant source) or fish oil (an animal source), can be used to improve bull fertility.

## Supporting information

S1 Data (XLSX)
